# The effect of cardiovascular risk factors on the longitudinal evolution of the carotid intima medial thickness in children with type 1 diabetes mellitus

**DOI:** 10.1186/1475-2840-10-53

**Published:** 2011-06-16

**Authors:** Robert Dalla Pozza, Andreas Beyerlein, Claude Thilmany, Claudia Weissenbacher, Heinrich Netz, Heinrich Schmidt, Susanne Bechtold

**Affiliations:** 1 Department of Pediatric Cardiology, Ludwig-Maximilians-University, Marchioninistr. 15, D-81377 Munich, Germany; 2 Division of Epidemiology, Institute of Social Pediatrics and Adolescent Medicine, Ludwig-Maximilians-University, Heiglhofstr. 4, D-81377 Munich, Germany; 3 Division of Endocrinology and Diabetology, University Children's Hospital, Lindwurmstr. 4, D-80337 Munich, Germany

**Keywords:** Intima medial thickness, subclinical atherosclerosis, Type 1 diabetes mellitus, Follow up study, cardiovascular risk factors

## Abstract

**Background:**

Type 1 diabetes mellitus is a generally accepted atherogenic risk factor. The aim of this prospective longitudinal study was to evaluate changes in carotid intima media thickness (cIMT) in children and adolescents with type 1 diabetes mellitus (T1DM) using standardized methods.

**Methods:**

We re-evaluated cIMT in 70 (38 f) of initial 150 (80 f) patients with T1DM after 4 years. At re-evaluation, mean (± SD) age was 16.45 ± 2.59 y, mean diabetes duration was 9.2 ± 3.24 y and patients had a mean HbA1c of 8.14 ± 1.06%.

**Results:**

Mean cIMT z-scores increased significantly during 4 years (0.58 ± 0.75, p < 0.001) as well as BMI-z-score (0.41 ± 0.81, p < 0.01), systolic blood pressure (0.77 ± 1.15, p < 0.01) and HbA1c (0.90 ± 1.07, < 0.001). In a linear regression model systolic blood pressure z-score at first measurement (0.02, CI: 0.01, 0.04) was a significant predictor for the mean effect on cIMT z-score. In a logistic regression model significant risk factors for an increase in IMT of ≥1.5 z-scores were BMI z-scores (OR: 3.02, CI:1.11, 10.14), diabetes duration (OR:1.32, CI:1.04, 1.77) and systolic blood pressure (OR: 1.14, CI: 1.04, 1.27) at first measurement each.

**Conclusions:**

Longitudinal cIMT measurements revealed progression in subclinical atherosclerosis during a four year period in diabetic children and adolescents. Systolic blood pressure and BMI were related to cIMT increment. Control of these risk factors by lifestyle and medical intervention may prevent progression of cIMT in diabetic children.

## Background

Cardiovascular disease as a result of macrovascular atherosclerotic changes is the major cause for mortality among patients with diabetes mellitus [1]. Even if these complications affect predominantly the adult diabetic patient, the process of vascular changes starts much earlier. Autopsies have shown that the atherosclerotic processes at the endothelial level begin in childhood and progress rapidly in the presence of risk factors [2]. Thus, children with diabetes mellitus are considered as high risk patients and special attention to vascular health has been recommended [3].

Common carotid artery intima-media thickness (cIMT) as measurable by high resolution B-mode ultrasonography is a non-invasive marker of subclinical atherosclerosis [4]. An increased IMT has been correlated to an increased relative risk for stroke and coronary arterial disease in adults [5]. In children, a significant thickening of the endothelial wall has been found in several chronic diseases including diabetes mellitus [6].

In the past a cross sectional study in 150 pediatric diabetic patients in our center revealed significantly increased cIMT values [7]. Age at disease onset was inversely, and HbA1c directly correlated to cIMT. A relatively small subgroup of these patients with increased cIMT was re-evaluated after a 2-year-interval to elucidate preliminary data on short-term cIMT progression [8].

Now we present the results of the 4-years prospective longitudinal cIMT assessment in the entire patient cohort and compared them with data on classical atherogenic risk factors available for the complete study period. We hypothesized that with age and diabetes duration cIMT progression take place.

## Methods

From a total of 150 eligible children and adolescents with type 1 diabetes mellitus included in the first cross sectional study, 70 patients could be enrolled for follow up. The majority of patients not included in this study was now cared for in the adult clinic and invited to take part in the study, but did not show up. To exclude a selection bias we compared the previous group of 150 patients with the remaining 70 patients with special attention to anthropometric and metabolic parameters. Patients enrolled only in the first measurement (n = 80, 40 m) had a significantly longer diabetes duration (7.37 ± 4.54 vs. 5.21 ± 3.29 years, p = 0.014), higher HbA1c (7.80 ± 1.06 vs. 7.22 ± 0.73%, p = 0.01) and a lower cIMT z-score (1.15 ± 0.58 vs. 1.82 ± 0.62, p = 0.01).

As reported in the first study, the diagnosis of diabetes mellitus was based on the current criteria of the American Diabetes Association. All patients were recruited consecutively during their regular 3-months visits as outpatients at our tertiary health care center (University Children's Hospital, Division of Endocrinology and Diabetology). None of the patients had evidence of retinopathy (as determined by dilated fundoscopy), clinical neuropathy or overt nephropathy. Medical records were available for all patients (data on HbA1c and insulin dosage as well as on diabetic complications such as microalbuminuria defined as three positive spontaneous urine samples during the follow up visits followed by a total of three positive 8-hours night-urine collections on consecutive days to confirm the diagnosis, reference range for albumin-creatinine ratio < 20 mg/g creatinine) for the entire follow up period. Pubertal stage was defined according to the criteria published by Tanner et al. [9]. Waist-to-hip ratio was calculated from the waist circumference and the hip circumference measured according to the WHO recommendations: Waist was measured at the level midway between the lower rib margin and the iliac crest. Hip was measured as the maximum circumference over the buttocks. Absolute values were transformed in age- and sex-dependent values. Written informed consent was obtained of all participants from their legal guardians. The study was performed according to the Declaration of Helsinki and the study protocol was approved by the local ethics committee.

## Ultrasonography

As reported previously, the ultrasonographic study was performed with the patient supine for at least 10 minutes in a quiet room at 22 degrees centigrade. For data acquisition, a Philips IE33 was used equipped with a linear 11.0 MHz transducer (Philips, Germany). All studies were done according to a standardized scanning protocol for the right and left common carotid arteries. The common carotid artery bulb was identified and the segments of the common carotid arteries 1 to 2 cm proximal to the bulb region were scanned. The image was focussed on the posterior - the far - wall. Two angles were used at each side for scanning the common carotid artery IMT: lateral and anterior-oblique [10]. Simultaneously, an ECG tracing was recorded. All images were obtained in the 1st and the 2nd cIMT measurement by the same single, experienced ultrasonographer blinded to the patients metabolic state. The images were stored digitally for subsequent offline-analysis. For the measurement of the cIMT, the distance in the still frame at the end of the diastole (R-wave of the ECG) between the leading edges of the lumen - intima interface and the media - adventitia interface of the B-mode frame was considered. Computer software (Qlab, Philips, Germany) which analyzed the cIMT-distance automatically at 64 points within a segment of 10 mm was adopted; the value given was the arithmetic mean cIMT calculated. A manual second reading of the accurate border detection during computed analysis was performed in all images obtained. The calculation of the mean cIMT was determined from 2 separate scans of each side; thus, the conclusive mean cIMT of each single patient was calculated from the 4 mean cIMT-values (a total of 256 points analyzed). In the 1^st ^and 2^nd ^cIMT measurement, we calculated an intraobserver variability of 2.5% and 2.1%, respectively. The coefficient of variation between the single measurements was 2.6%. In the 1st measurement, we investigated also a control group (n = 58) to assess the quality of our measurement results and found no significant differences to normal values from the literature. For the calculation of z-scores of cIMT, the sex- and height-dependent normative values from the literature were adopted [11].

## Blood pressure measurements

Blood pressure measurement during the regular 3-months-follow-up visits was performed in a quiet room. Blood pressure was obtained using a conventional oscillatory measurement system positioned at the right upper arm (Dinamap, GE Systems, Germany). Measurements of blood pressure were performed for each clinic visit (3.2 ± 0.4 visits per year) and averaged for one year. Z-scores were calculated adopting normative values from the literature [12].

## Laboratory Methods

Fasting blood samples were taken during the patients` follow up visit. Total cholesterol, high density (HDL) and low density cholesterol (LDL) were measured by standard laboratory methods. Each sample was processed immediately following the patient's visit with a maximum delay of one hour. Serum leptin levels were measured in duplicate by RIA using commercial kits (Human leptin RIA, Linco Research Inc., St. Charles, MO, USA). The limit of sensitivity was 0.5 μg/L, the intraassay CV was 8.2%, and interassay CV was 6.5%. Adiponectin was measured by ELISA (Mediagnost, Reutlingen, Germany). The limit of sensitivity was <0.6 μg/L, the intraassay and interassay CV was < 8.5% and < 5.4%, respectively. The HbA1c level was measured by DCA 2000 ™, based on specific inhibition of latex immunoagglutination (Bayer AG, Leverkusen, Germany). Normal values of HbA1c as established in our laboratory range from 4.0% to 6.0%. Moreover, an average HbA1c was calculated for each patient taking the mean of measurements (3.2 ± 0.4 visits per year) during the previous 12 months before each cIMT measurement.

## Statistics

Calculations were performed using the statistical package SPSS for Windows (version 14.0, SPSS, Chicago, IL, USA) and R 2.12.1 (http://cran.r-project.org). Differences within the patient group and at different time points were examined by the non-parametric Mann-Whitney U-test. Statistical significance was defined as p < 0.05, 2-sided. Spearman's rank correlation was used to determine r values. We performed a linear regression analysis to evaluate the associations between mean cIMT z-scores differences between the two measurements (dependent variable) and BMI z-scores, mean HbA1c, diabetes duration, insulin dose, LDL cholesterol, HDL cholesterol and systolic blood pressure (independent variables) at first and second measurement (in separate models). We further performed a logistic regression analysis to identify predictor variables for an increase ≥ 1.5 SD in cIMT of over time. Regression analyses were performed in observations with complete information on the dependent and all independent variables at each measurement (n = 61). In each model, backward variable selection was applied using the Akaike Information Criterion (AIC) (13).

## Results

Longitudinal data of 70 (32 m) patients could be analyzed. The characteristics of the study group at first cIMT measurement and 4 years later are reported in Table 1. Mean age at onset of type 1 diabetes was 7.36 ± 3.78 years. All patients were on intensive insulin treatment (n = 51) or on insulin pump therapy (n = 19).

**Table 1:** Characteristics of the study population of patients with Type 1 diabetes mellitus

	**1**^**st **^**cIMT measurement (n = 70)**	**2**^**nd **^**cIMT measurement (n = 70)**	p-value of change
Age (years)	12.61 ± 2.49	16.45 ± 2.59	<0.001
Diabetes duration (years)	5.20 ± 3.31	9.21 ± 3.24	<0.001
Height (cm)	156.21 ± 16.51	170.1 ± 11.83	<0.001
Weight (kg)	50.58 ± 16.68	67.57 ± 14.79	<0.001
BMI (kg/m^2^)	20.15 ± 3.56	23.17 ± 3.53	<0.001
BMI z-score	0.45 ± 0.94	0.86 ± 1.10	<0.001
Waist hip ratio	n.a.	0.57 ± 1.40	
Systolic blood pressure (mmHg)	109.27 ± 10.68	122.47 ± 11.50	<0.001
Systolic blood pressure z-score	0.59 ± 0.80	1.37 ± 1.05	<0.001
LDL-cholesterol (mg/dl)	88.30 ± 24.71	85.87 ± 27.75	0.43
HDL-cholesterol (mg/dl)	60.04 ± 15.61	63.25 ± 15.43	0.085
HbA1c (%)	7.24 ± 0.74	8.14 ± 1.06	<0.001
Insulin dose (IU/kg body weight/day)	0.81 ± 0.32	0.98 ± 0.26	<0.001
Microalbuminuria (number of patients)	4	5	0.322
Antihypertensive treatment (number of patients)	1	10	0.002
Smoking (number of patients	1	10	0.004
cIMT (mm)	0.469 ± 0.031	0.478 ± 0.031	<0.001
cIMT z-score	1.86 ± 0.61	2.44 ± 1.00	<0.001

Data are means ± SD; cIMT carotid intima medial thickness; BMI body mass index; LDL low density lipoprotein cholesterol; HDL high density lipoprotein cholesterol.

At first cIMT measurement 28 (17 m) patients were prepubertal, 42 (15 m) patients in early puberty corresponding to Tanner stage 2 and 3. Patients proceeded with pubertal development, at second measurement 28 (16 m) patients were in early or mid puberty (Tanner stage 2 or 3), whereas 42 (16 m) patients were late or postpubertal (Tanner stage 4 or 5 ).

During the follow up period patients had a significant increase in mean BMI z-score (0.41, CI: 0.61, 0.21) and mean systolic blood pressure z-score (0.77, CI: 1.06, 0.48). Regarding the metabolic profile, there was a significant increase in mean HbA1c levels (0.90%, CI: 0.63, 1.17), whereas HDL-cholesterol (2.89 mg/dl, CI: -0.41, 6.2) and LDL-cholesterol remained stable (-2.60 mg/dl, CI: -9.1, 3.9).

### cIMT measurement

**Figure 1:**
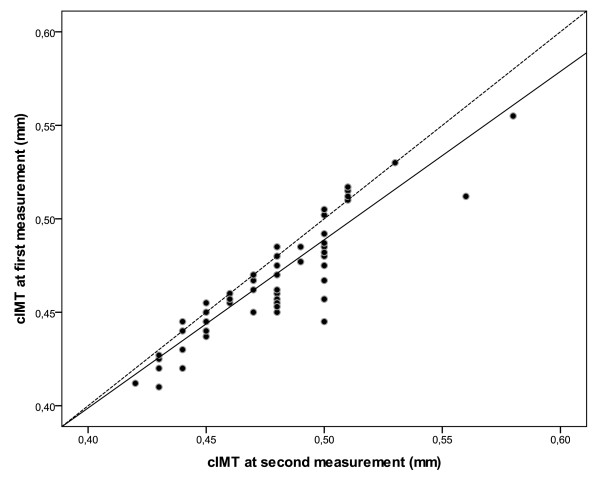
**Close association of cIMT measurements at baseline and after 4 years, solid line represents the fit line at total and broken line represents the reference line from the equation y = x**.

At first measurement, cIMT-z-score correlated inversely with age at diabetes manifestation, (r = -0.42, p = 0.002). At second measurement, cIMT correlated with the level at 1st measurement: the higher cIMT z-score at first measurement, the higher cIMT-z-score at 2^nd ^measurement (r = 0.63, p < 0.001) (Figure 1). There was no difference between male and female patients at first and second cIMT measurement. A major increase of ≥ 1.5 SD z-scores over 4 years was developed in 14.5% of patients (Table 2). Significant differences between the high increase group and the remaining study population included diabetes duration and systolic blood pressure at first evaluation. Separating patient groups at the 2nd cIMT measurement according to Tanner stages in early- or midpubertal (n = 28) and late or postpubertal patients (n = 42), those more advanced in pubertal development had a much higher mean increase in cIMT-z-scores than younger patients (0.80 ± 0.70 vs. 0.27 ± 0.70 SD, p < 0.05).

**Table 2:** Characteristics of patients with a major increase in IMT z-scores of ≥ 1.5 SD over 4 years in comparison to the remaining patients

	cIMT- z-score increase ≥1.5 SD (n = 11)	cIMT z-score increase < 1.5 SD (n = 55)	p-value of mean difference
Age_2 (at 2^nd ^cIMT measurement) (years)	17.59 ± 2.87	16.08 ± 2.48	0.078

Age at disease manifestation (years)	6.97 ± 4.02	7.44 ± 3.77	0.71

Diabetes duration_2	10.85 ± 3.92	8.77 ± 2.93	**0.047**

Puberty_2 (early-mid pubertal/late or postpubertal)	2/9	25/30	0.096

Microalbuminuria and ACEi treatment_2 (patients)	4	11	0.42

Smoking_2 (patients)	2	8	0.741

HbA1c_1 (1^st ^measurement) (%)	6.99 ± 0.51	7.27 ± 0.77	0.250

HbA1c_2 (%)	7.60 ± 0.77	8.23 ± 1.08	0.085

LDL-cholesterol_2 (mg/dl)	84.27 ± 23.11	86.73 ± 28.66	0.79

HDL-cholesterol_2 (mg/dl)	65.27 ± 16.30	62.69 ± 15.52	0.62

Leptin (μg/L)	11.51 ± 6.32	8.08 ± 8.03	0.219

Adiponectin (μg/L)	12.69 ± 5.74	13.54 ± 4.13	0.619

Systolic blood pressure_1 (mmHg)	116.50 ± 8.86	107.81 ± 10.31	**0.011**

Systolic blood pressure_1 z-score	0.99 ± 0.95	0.49 ± 0.74	0.056

Systolic blood pressure_2 (mmHg)	128.20 ± 12.30	121.41 ± 11.15	0.086

Systolic blood pressure_2 z-score	1.77 ± 1.28	1.29 ± 1.03	0.192

BMI_1 z-score	0.82 ± 0.95	0.33 ± 0.88	0.796

BMI_2 z-score	0.91 ± 1.03	0.82 ± 1.10	0.103

cIMT-1 z-score	2.13 ± 0.48	1.81 ± 0.62	0.578

cIMT-2 z-score	3.83 ± 0.43	2.16 ± 0.84	**<0.001**

Data are means ± SD; cIMT carotid intima medial thickness; ACEi angiotensin converting enzyme inhibitor; LDL low density lipoprotein cholesterol; HDL high density lipoprotein cholesterol; BMI body mass index.

### Classical cardiovascular risk factors

Looking for traditional cardiovascular risk factors we compared normal weight (n = 44) and overweight diabetic patients (BMI z-score >1.28, n = 26) at 2nd measurement and found no difference in age, sex distribution, systolic blood pressure or cIMT-z-score. In the group of patients with adiposity (BMI z-score >2, n = 13) there were more smokers (n = 5; 33%) and a significant difference in terms of systolic blood pressure z-scores (1.22 ± 0.9 vs. 2.06 ± 1.38, p = 0.014) and its increase over time (0.59 ± 1.02 vs. 1.73 ± 1.43, p = 0.004) as well as the number of patients on ACE inhibitor treatment (5/13 vs.5/57). The increase in BMI-z-score in the whole group of patients had no significant influence on cIMT development, but was associated with a higher increase in systolic blood pressure z-score (r = 0.44, p = 0.01). Leptin and adiponectin had no influence on cIMT development. At second measurement 16 patients (23%) were hypertensive. Within this group 60% were male and 27% were smokers, whereas in the normotensive group were 10% smoker. Patients with hypertension had a significantly higher cIMT z-score (0.67, CI: 0.09, 1.24).

In a multivariable linear regression model for the mean effect on cIMT z-score change HbA1c (effect per%: -0.29, CI: -0.53, -0.05) and systolic blood pressure z-score at first measurement (0.02, CI: 0.01, 0.04) remained as significant predictors after backward selection. With respect to variables from second measurement, the only remaining significant predictor for mean cIMT z-score change was systolic blood pressure z-score (0.02, CI: 0.00, 0.03) (data not shown). In logistic regression models significant risk factors for an increase in cIMT of ≥ 1.5 z-scores were at first measurement BMI z-scores, diabetes duration and systolic blood pressure, while at second measurement only systolic blood pressure was borderline significant (OR: 1.07, CI: 1.00, 1.15, p = 0.05)(Table 3).

**Table 3:** Linear regression on mean change in cIMT and logistic regression model on the risk to develop a major increase in cIMT z-scores of ≥ 1.5:

Prediction based on parameters at first measurement			Prediction based on parameters at first measurement		
***Full model linear regression***			***Full model logistic regression***		

**Predictor**	**OR [95%-CI]**	**p-value**	**Predictor**	**OR [95%-CI]**	**p-value**

BMI z-scores	0.04 [-0.17, 0.26]	0.7	BMI z-scores	2.78 [0.97, 9.69]	0.07

Mean HbA1c	-0.26 [-0.51, -0.01]	0.04	Mean HbA1c	0.33 [0.06, 1.36]	0.15

Diabetes duration	-0.02 [-0.08, 0.04]	0.47	Diabetes duration	1.40 [1.07, 2.00]	0.03

Insulin dose	-0.34 [-0.93, 0.24]	0.24	Insulin dose	0.46 [0.03, 6.45]	0.57

LDL-cholesterol	0.00 [-0.01, 0.01]	0.71	LDL-cholesterol	1.02 [0.99, 1.06]	0.28

HDL-cholesterol	-0.01 [-0.02, 0.00]	0.17	HDL-cholesterol	0.99 [0.92, 1.05]	0.65

Sys. blood pressure	0.02 [0.01, 0.04]	<0.01	Sys. blood pressure	1.15 [1.04, 1.31]	0.01

*Selected variables according to AIC*			*Selected variables according to AIC*		

HDL-cholesterol	-0.01 -[0.02, 0.00]	0.14	Mean HbA1c	0.29 [0.04, 1.11]	0.10

Mean HbA1c	-0.29 [-0.53, -0.05]	0.02	Diabetes duration	1.32 [1.04, 1.77]	0.03

Sys. blood pressure	0.02 [0.01, 0.04]	0.01	Sys. blood pressure	1.14 [1.04, 1.27]	<0.01

			BMI z-score	3.02 [1.11, 10.14]	0.046

BMI body mass index; LDL low density lipoprotein cholesterol; HDL high density lipoprotein cholesterol; sys systolic; AIC Akaike Information Criterion

## Discussion

In the present study on longitudinal evolution of carotid intima-media-thickness in children and adolescents with type 1 diabetes mellitus over 4 years we observed a significant progression in carotid IMT z-scores. We assume a promoting and accelerating effect of known cardiovascular risk factors on cIMT.

Macrovascular disease leading to cardiovascular complications remains the endpoint of type 1 diabetes mellitus in adulthood [1]. Atherosclerotic lesions develop slowly, but continuously leading to the main causes of mortality in diabetic patients such as coronary arterial disease, stroke, and renal failure [13]. In diabetic children, the presence of subclinical atherosclerotic disease as a precursor of macrovascular complications has been shown in several studies [6]. Also, impaired endothelial function preceding atherosclerotic changes has been observed [14]. As the measurement of the intima-media thickness of the carotid arteries is considered a surrogate marker of subclinical atherosclerosis, this method has been used widely in these patients to asses vascular health [6]. According to the results of several cross-sectional studies, evidence for an increased cIMT and aortic IMT in diabetic children emerged [6,15]. Data from observations on the cIMT in adult patients indicated that cIMT values remain stable or decrease when elevated cardiovascular risk factors like hypercholesterolemia, glycemic control or obesity are optimized [16]. This has been shown also in selected pediatric patient groups including children with hypercholesterolemia or overweight [16,17]. Even if recently a metaanalysis raised concerns on the ability of cIMT regression to predict reduction of cardiovascular events, several research groups recommended longitudinal cIMT assessment [18].

In a previous cross sectional study in children and adolescents with type 1 diabetes, we were able to show that cIMT is elevated in comparison to a healthy control group and to normative data. We proposed longitudinal cIMT measurements to elucidate more details on the individual cardiovascular risk based on the cIMT evolution [7]. Subsequently, we followed a selected subgroup of diabetic patients with elevated cIMT over 2 years [8]. Now, we present the results of a 4-year longitudinal prospective study in a cohort of 70 patients.

Within the patient group, we observed an individual increase of cIMT z-scores over 4 years. Thus, not only vascular health, but also vascular aging seems to be affected in diabetic children [19]. In concordance with adult patients data, also in diabetic children endothelial changes seem to be accelerated compared to normal values [20]. Interestingly, patients with elevated cIMT at baseline were more prone to accelerated vascular aging during the follow up period.

The association between cIMT and diabetes duration and age at manifestation was found by us and by other research groups [13,21]. Conversely, one group observed only aging, but not diabetes duration as a relevant factor for IMT increase[22]. Longer disease duration early in life might enhance the metabolic effects of diabetes on the vascular system and result in earlier onset and accelerated progression of atherosclerosis. However, with increasing age other classical cardiovascular risk factors may play an additional role in diabetic children. We observed an association of systolic blood pressure, diabetes duration and BMI with mean increase in IMT z-scores in linear regression analysis: For example, an increase in systolic blood pressure by 1 mmHg was associated with a mean increase in IMT-z-scores of 0.02. Further, children with longer disease duration had increased risk to show a major increase in cIMT (36% per year). Even more impressive was the influence of the BMI z-score at first measurement, increasing the risk to develop a major augmentation in cIMT z-scores by the factor 3.02. As resulting from the literature, the association of several cardiovascular risk factors seems to add deleterious effects to the vascular bed in diabetic children [23]. Recently, also postprandial glucose levels have been found to be correlated to cIMT [24]. Thus, current recommendations for the medical care of diabetic children include lower thresholds for blood pressure and BMI compared to healthy children [3]. Also in non-diabetic adolescents, the combination of systolic blood pressure, LDL-cholesterol, smoking and BMI measured at the age of 12 to 18 years were associated with adult cIMT [25]. Surprisingly, HbA1c was negatively associated with cIMT increment. This might be explained by a possibly higher rate of hypoglycemia and their deleterious effect on the endothelium. According to the recent publication by Wright et al. hypoglycemia triggers the expression of vasoactive substances (CD 40, soluble CD 40 ligand), markers of inflammation, thrombosis and endothelial dysfunction [26].

Within the study group, patients developed higher BMI z-scores. This is in accordance with other studies on weight and BMI development in children and adolescents with diabetes type 1 [23]. The BMI as mentioned above played a major role in cIMT development. In the entire group, systolic blood pressure, LDL z-scores, HbA1c levels, and insulin requirement progressed. Also, the number of smokers increased. As known from adult patients, components of the metabolic syndrome contribute to accelerated early atherosclerosis. Leptin and adiponectin levels, though elevated throughout the study group, failed to show any correlation to cIMT values. Thus, in our patients, these hormones seem not to play a key role as mediators of vascular damage in visceral adiposity. So, we may not propose to investigate on leptin and adiponectin levels in diabetic children for the assessment of vascular health so far [27].

The majority of patients showed a cIMT z-score increment during pubertal development. This is in accordance with Wiegman et al. who found a significant deviation in cIMT at the age of 12 years in children with familial hypercholesterolemia compared to unaffected siblings [28]. Adolescence has been identified as a critical time in determining risk factors of future vascular complications in type 1 diabetes mellitus [29]. Interventions to prevent these complications may be most efficient if implemented at a young age before puberty. Early cIMT measurement possibly could help to identify diabetic patients at risk to develop cIMT increment, followed by individualized care to optimize the metabolic profile (e.g. BMI) and blood pressure during puberty. Interestingly, we could not find gender differences in cIMT values in our patients. We speculate that the influence of sexual hormones on the cIMT is minimal in adolescence and early adulthood; supported by the fact that normal values on cIMT in this age group are similar for males and females [11].

Some limitations of our study have to be addressed. An observational study cannot establish causality. Our measurement method differs from some other research groups; but according to the actual recommendations semi automated border detection software is the best solution for cIMT measurement up to date. Also, the normative data adopted were obtained by the same method and protocol [11]. Furthermore, we were able to demonstrate excellent intra-observer reliability for our cIMT measurement. A further limitation is the loss-to-follow-up of 80 pts. However, the presented cohort is representative in number and may show more pronounced influencing factors, though it had higher cIMT z-scores initially.

## Conclusion

Concluding, the results of our study indicate that children and adolescents with type 1 diabetes mellitus show increased cIMT values also after a period of 4 years. Patients with increased cIMT at baseline show a further acceleration in cIMT increment indicating that vascular changes continue to evolve throughout puberty and adolescence. In a multivariate logistic regression model systolic blood pressure, diabetes duration and BMI were associated with major cIMT increment. So, we propose to optimize blood pressure levels and BMI development as early as possible in diabetic children to prevent long-term vascular damage. Further studies addressing the effect of interventional programmes on the cIMT are warranted.

## Authors declaration on Competing interests

The authors declare that they have no competing interests.

## Authors' contributions

RDP contributed in research design, performance, calculations and writing. AB contributed in research calculations and writing. CT contributed in research performance. CW contributed in research performance. HN contributed in research design and results interpretation. HS contributed in research design and reults interpretation. SB contributed in research design, performance, calculations, results interpretations and writing. All authors read and approved the final version of this manuscript.
